# TRPM3, TRPM4, and TRPM5 as thermo-sensitive channels

**DOI:** 10.1186/s12576-024-00937-0

**Published:** 2024-09-18

**Authors:** Kunitoshi Uchida

**Affiliations:** 1 Laboratory of Functional Physiology, Department of Environmental and Life Sciences, School of Food and Nutritional Sciences, University of Shizuoka, Yada 52-1, Suruga-Ku, Shizuoka, Shizuoka 422-8526 Japan; 2 Graduate School of Integrated Pharmaceutical and Nutritional Sciences, University of Shizuoka, Shizuoka, Japan

**Keywords:** TRP channel, Thermo-sensitivity, TRPM3, TRPM4, TRPM5

## Abstract

Temperature detection is essential for the survival and perpetuation of any species. Thermoreceptors in the skin sense body temperature as well as the temperatures of ambient air and objects. Since Dr. David Julius and his colleagues discovered that TRPV1 is expressed in small-diameter primary sensory neurons, and activated by temperatures above 42 °C, 11 of thermo-sensitive TRP channels have been identified. TRPM3 expressed in sensory neurons acts as a sensor for noxious heat. TRPM4 and TRPM5 are Ca^2^⁺-activated monovalent cation channels, and their activity is drastically potentiated by temperature increase. This review aims to summarize the expression patterns, electrophysiological properties, and physiological roles of TRPM3, TRPM4, and TRPM5 associated with thermosensation.

## Introduction

Temperature detection is essential for the survival and perpetuation of any species. In homeotherms, maintaining core body temperature within a narrow range across a wide range of ambient temperatures is a fundamental aspect of life. Thermoreceptors in the skin sense the temperature of the ambient air and objects touched. Thermal nociception is triggered both by extreme temperatures and strong mechanical stimuli. One group of thermal nociceptors is excited by noxious heat (temperatures above 45 °C), and the second group responds to noxious cold (temperatures that cool the skin below 5 °C). Innocuous temperature changes can be detected in the skin and/or in primary sensory neurons. Primary sensory neurons detect and transmit temperature information to the brain, with one type innervating the skin or viscera and another projecting to the dorsal horn of the spinal cord or the spinal trigeminal nucleus in the brainstem. Somatosensory temperature information is transmitted to the thalamus via the anterolateral system. The axons that cross the midline of the medulla to form the medial lemniscus are called second-order neurons. As these axons ascend through the brainstem, they shift laterally, joining the fibers of the spinothalamic tract in the midbrain. Third-order neurons innervate primary somatosensory areas of the cerebral cortex. Temperature information is also transmitted to the preoptic area (POA) of the hypothalamus, which is the center of unconscious autonomic control of body temperature [44]. Changes in the internal temperature of the brain are detected by specialized thermoreceptors located throughout the core of the body, including the viscera, brain, and spinal cord, with the most sensitive site being the POA. Local heating or cooling can induce feedback responses to maintain a narrow range of desired body temperatures.

In 1997, Julius et al. identified a receptor expressed in small-diameter primary sensory neurons activated by capsaicin. They also discovered that this receptor is activated at temperatures above 42 °C [10]. To our knowledge, this is the first study identifying thermal receptors in primary sensory neurons. This receptor was initially named vanilloid receptor 1 (VR-1). Given that VR-1 belongs to the transient receptor potential superfamily, it was renamed transient receptor potential vanilloid 1 (TRPV1). Since their discovery, researchers have identified more ion channels with high thermo-sensitivity, and many of these proteins belong to the TRP family. Evidence that ion channels act in temperature sensations is supported by their high temperature coefficients (Q_10_). This parameter describes the rate of change of a chemical or biological reaction when temperature is increased by 10 °C. As this increase in temperature usually results in the reaction rate doubling, the Q_10_ value of the reaction is 2. Although the Q_10_ values of most ion channels are approximately 2 [87], thermo-sensitive ion channels exhibit much higher Q_10_ values.

**Fig. 1:**
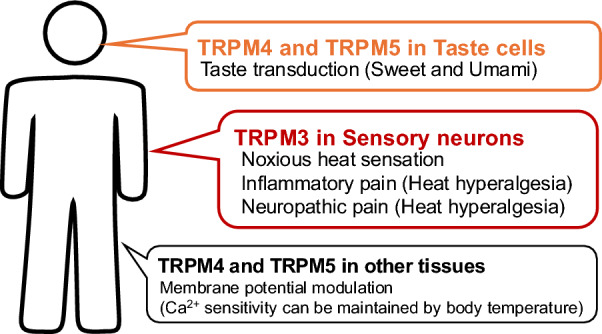
The physiological roles of TRPM3, 4 and 5 channels related to thermo-sensation

Most transient receptor potential (TRP) channels are non-selective cation channels. In mammals, TRP channels are divided into six subfamilies: TRPC (canonical), TRPV (vanilloid), TRPM (melastatin), TRPML (mucolipin), TRPP (polycystin), and TRPA (ankyrin). To date, 11 TRP channels have been reported to exhibit thermo-sensitivity and are hence called thermo-sensitive TRP channels. These channels belong to the TRPV, TRPM, TRPA, and TRPC subfamilies, and their activation temperature thresholds are within the range of physiological temperatures. TRPV1, TRPV2, and TRPM3 are activated at elevated temperatures, whereas TRPM8 and TRPC5 are activated at cool and cold temperatures. Although TRPA1 has also been reported to be activated by cold temperatures, its thermo-sensitivity remains controversial. TRPV3, TRPV4, TRPM2, TRPM4, and TRPM5 are all activated by warm temperatures [29, 56]. Thermo-sensitive TRP channels function as "multimodal receptors" that respond to various chemical and physical stimuli. For example, TRPV1, which is activated by noxious heat, is also a receptor for several pungent agents, such as capsaicin, as well as low pH [73]. Activation of these channels could contribute to changes in intracellular Ca^2+^ concentrations and control membrane potentials in many cell types. Thermo-sensitive TRP channels are expressed in sensory neurons, and the skin can act as an ambient temperature sensor [72]. Additionally, thermo-sensitive TRP channels are expressed in tissues that are not exposed to dynamic temperature changes, suggesting that these channels can sense internal temperatures or exhibit physiological functions unrelated to temperature detection. This review provides an overview of the thermo-sensitivity and associated physiological functions of the TRPM3 channel, which acts as a heat sensor. Additionally, it outlines the thermo-sensitivity of TRPM4 and TRPM5 channels, which belong to the melastatin subfamily TRPM3 (Fig. 1).

## TRPM3

TRPM3, a molecular determinant that acts as a store-operated calcium entry protein, is a member of the melastatin subfamily of TRP channels and comprises a nonselective cation channel, similar to other TRP channels. TRPM1, the protein most similar to this novel TRP gene, was used as a template to predict additional exons in TRPM3 from the same genomic sequence [36]. Ion selectivity, measured as permeability ratios, of mTRPM3 are 1.14 for PCa/PCs, 0.74 for PNa/PCs, and 1.57 for PCa/PNa [19]. The prevalent expression pattern of TRPM3 was detected in the kidney and at lower levels in the central and peripheral nervous systems and testis. Northern blot analysis of multiple mouse tissues revealed strong signals in the brain, whereas no signal was detected [19, 36]. RT-qPCR indicated the strongest expression of TRPM3 in the brain, pituitary gland, kidney, dorsal root ganglia, and adipose tissue but low abundance or absence of TRPM3 transcripts in other tissues [16, 28, 34]. In addition, other reports have demonstrated that TRPM3 is expressed in the ovaries, pancreas, retina, inner ear, and heart and is also expressed in cerebellar Purkinje neurons [89] and oligodendrocytes [52] within the brain.

The endogenous activators of TRPM3 are pregnenolone sulfate, a neuron steroid, and D-erythro-sphingosine [23, 83, 85]. Steroids (dihydroepiandrosterone, progesterone, testosterone, and estradiol) are partial agonists of TRPM3 [57]. Additionally, TRPM3 activity alters extracellular osmolarity [19]. Drugs with other targets, including nifedipine and clotrimazole, are known to also activate the TRPM3 channel [83, 85]. TRPM3 activation is inhibited endogenously by the G protein βγ subunits [5, 12, 60] and by diclofenac via pregnenolone sulfate and nifedipine [67]. CIM0216 is a synthetic ligand of TRPM3 [21]. Volatile anesthetics (e.g., chloroform, halothane, isoflurane, and sevoflurane) inhibit TRPM3 via pregnenolone sulfate, CIM0216, and temperature elevation [30]. Rosiglitazone and other peroxisome proliferator-activated receptor-γ (PPARγ) agonists (troglitazone and pioglitazone) also inhibit TRPM3 activation by pregnenolone sulfate and nifedipine [42].

In 2011, it was reported that TRPM3 is expressed in a subset of primary sensory neurons and is a temperature sensor that is activated at approximately 37 °C [84]. The TRPM3α2 variant was used for functional analysis. In a heterologous expression system, the Q_10_ value of heat-evoked current of TRPM3α2 was 7.2 and there was no activation threshold from 25 to 40 °C [84]. The weakly temperature-dependent activation of purified TRPM3α2 protein was observed in artificial lipid bilayer membranes, and the Q_10_ value of open probability (channel activity) was 5 [75]. In planar lipid bilayer experiments, the temperature dependence of TRPM8 was Q_10_ = 40, whereas that for TRPV1 was Q_10_ = 18 [66, 88]. In heterologous expression experiments, the Q_10_ for both TRPV1 and TRPM8 was more than 10 [8, 82], indicating that in some experimental settings, the differences between these TRP channels are not large. In the presence of phosphatidylinositol 4,5-bisphosphate (PIP_2_), TRPM3α2 exhibited a Q_10_ of 5.3, which is relatively close to that observed in cells (Q_10_ = 7.2) [84]. In contrast, in *Xenopus* oocyte recordings, the Q_10_ of current for TRPM3α2 was 55, and the activation temperature threshold was 40 °C [76]. As concluded previously, some molecules, including PIP_2_, may be necessary for temperature-dependent TRPM3 activation [75], although some of these candidate molecules may also exist in *Xenopus* oocytes.

In heterologous expression systems, the single-channel conductance of TRPM3 is 50–133 pS. One report indicated that the single-channel cord conductance was 133 pS for 140 mM Cs^+^, 83 pS for 140 mM Na^+^, and 65 pS for 100 mM Ca^2+^ [19]. Another report demonstrated that the conductance of TRPM3 stimulated by pregnenolone sulfate was 87 pS [67]. Vriens et al. provided evidence for a second permeation pathway in the TRP channel TRPM3, which can be gated by the combined application of pregnenolone sulfate and exogenous chemicals, such as clotrimazole. That is, the conductance of TRPM3 by pregnenolone sulfate is approximately 50 pS, and the unitary amplitude of TRPM3 by clotrimazole is 2 pA at -150 mV (~ 13 pS) [83]. In bilayer lipid membrane, when the concentrations of PIP_2_ and pregnenolone sulfate were increased up to 10 and 15 μM, we observed the TRPM3 opening with small conductance (less than 10 pS) [75]. A conductance larger than 10 pS was defined as large conductance, and a conductance smaller than 10 pS was defined as small conductance. The inward currents from the large conductance region exhibited a mean slope conductance level of 15 pS. Outward currents from large conductance were observed, with a main slope conductance value of 23 pS. The low conductance exhibited a linear I–V relationship, and the slope conductance was 5.9 pS. The Q_10_ of conductance with nifedipine was less than 2, indicating that the conductance of TRPM3α2 is not significantly affected by temperature increases [75]. Temperature elevation profoundly affects the opening probability of the TRPM3 channel, thereby strongly influencing its gating.

It is well known that PIP_2_ regulates the activity of many ion channels, including TRP channels. PIP_2_ was not necessary for the activation of TRPM3α2 by nifedipine, but PIP_2_ was necessary for activation by pregnenolone sulfate [75]. Directly agonistic action of pregnenolone sulfate on TRPM3 gating was observed, although pregnenolone sulfate alone was insufficient and required a cofactor for its activity. Hence, pregnenolone sulfate-induced TRPM3 opening occurred only in the presence of PIP_2_ or clotrimazole. TRPM3 regulation by phosphoinositides has similarly been observed in patch-clamp recordings [4, 74], indicating a physiological role in channel activity. While temperature increases (≤ 42 °C) did not induce any noticeable channel activity of purified TRPM3α2 in bilayer lipid membrane, application of PIP_2_ is sufficient to activate TRPM3 by temperature increases [75]. Conversely, the addition of pregnenolone sulfate during the temperature test was ineffective to activity of purified TRPM3α2 protein in an artificial bilayer lipid membrane [75]. Additionally, temperature elevation did not affect TRPM3 activity induced by nifedipine in an artificial bilayer lipid membrane. In the heterologous expression of TRPM3 in HEK293 cells, temperature dependence was notably enhanced in the presence of pregnenolone sulfate [84], suggesting that the modulation of TRPM3 activation by molecules in living cells, including calmodulin, could enhance the temperature sensitivity of TRPM3 [25, 59].

**Fig. 2:**
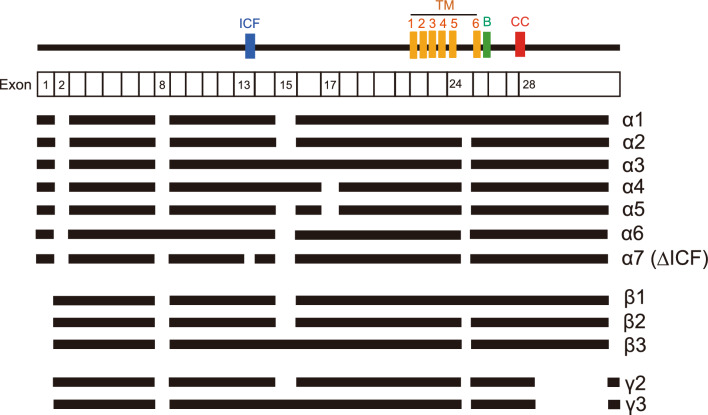
The topology of TRPM3 variants. The upper line indicates the membrane topology model of TRPM3, and black bars for each variant indicate coding regions. *ICF* indispensable for channel function region, *TM* transmembrane domain, *B* TRP box, *CC* coiled-coil domain. This figure is a modification of Fig. 1 from the paper written by Uchida et al. [76]

TRPM3 has many variants, more than 10 of which have been reported (Fig. 2). These variants can be divided into two main groups depending on the presence of deletions in exons 1, 2, or 28, which contain the start or stop codon [17]. However, functional analyses of TRPM3 variants have been largely limited to these α variants. Only a few features of TRPM3 variants are reported, and some reports demonstrated the functional differences TRPM3α1, α2, and α7. With 12 amino acid residues inserted in the pore loop domain, TRPM3α1 showed low permeability for Ca^2+^ and other divalent cations compared to TRPM3α2, which lacks these 12 amino acid residues. [51]. TRPM3α7 lacks the 10 amino acid residues known as the ICE region in exon 13 (TRPM3ΔICF), and this TRPM3ΔICF variant exhibits reduced interaction with other TRPM3 isoforms as well as reduced localization to cell membranes [17]. TRPM3ΔICF has been reported to be a non-functional channel, and the expression level of TRPM3ΔICF differs among tissues. TRPM3α2-6 variants were activated by pregnenolone sulfate and by nifedipine, whereas the long pore loop variant TRPM3α1 was insensitive to either compound. In contrast, TRPM3α1 was robustly activated by clotrimazole, a compound that does not directly activate the short pore variants but potentiates their responses to pregnenolone sulfate. In contrast, TRPM3α1 is insensitive to both hypoosmotic and heat stimulation [20].

TRPM3 variant 6 is very different from other variants in that it lacks the 1169 bp sequence in exon 28 and splice 23 bp sequence in the rest of exon 28, known as the γ subtype [76]. TRPM3γ variants had low activation relative to the TRPM3α2 variant in response to pregnenolone sulfate and nifedipine in HEK293 and *Xenopus* oocytes. This low activation may be due to reduced protein expression (including membrane expression) and/or impaired channel activity [76], because the potency of the TRPM3γ in response to pregnenolone sulfate treatment was much lower than that of the TRPM3α2 variant. A study by Vriens et al. revealed that both HEK293 cells expressing TRPM3α2 and dorsal root ganglion (DRG) neurons showed TRPM3-dependent activation in response to increased temperature [84]. Another study reported that TRPM3α2 protein reconstituted into lipid bilayers exhibits diminished temperature dependence [75]. As described above, in *Xenopus* oocyte recordings, the Q_10_ of the activating current for TRPM3α2 during heat stimulation was 30–55 and the activation temperature threshold was 40 °C [76]. Conversely, TRPM3γ variants had low activation relative to the TRPM3α variants in response to temperature elevation in *Xenopus* oocytes.

In addition, the mRNA expression level of TRPM3γ variants was significantly higher than TRPM3α variants, and lower than total expression of TRPM3 variants (α + β + γ) in mouse DRG, suggesting that the mRNA expression level of TRPM3α variants is lower than that of TRPM3β variants. TRPM3 is expressed in DRG and trigeminal (TG) neurons. DRG and TG neurons were classified by Vriens et al. using Ca^2+^imaging experiments. The population of pregnenolone sulfate-sensitive neurons comprised approximately 58% of DRG neurons and 57% of TG neurons. Pregnenolone sulfate-sensitive neurons are mainly small-diameter neurons and are almost completely abolished in TRPM3 knockout neurons [65, 84]. Wild-type mice show heat sensitivity, with 82% of DRG neurons and 79% of TG neurons responding to heat stimulation, which is slightly reduced in TRPM3 knockout sensory neurons, with 59% of DRG neurons and 63% of TG neurons responding to heat [84]. Heat-sensitive neurons were classified into three groups based on a pharmacological study using pregnenolone sulfate (a TRPM3 agonist) and capsaicin (a TRPV1 agonist). Among heat-positive wild-type sensory neurons, 43% were TRPM3- and TRPV1-expressing neurons and 33% were only TRPM3-expressing neurons [84]. Tan et al. showed a similar characterization of DRG neurons. The population of neurons sensitive to both capsaicin and heat was 28%; sensitivity to both pregnenolone sulfate and heat was 31%; and sensitivity to capsaicin, pregnenolone sulfate, and heat was 13% [71]. In particular, the number of heat-sensitive neurons responding to pregnenolone sulfate but not to capsaicin was strongly reduced in TRPM3 knockout mice [84].

A 2011 study by Vriens et al. demonstrated that TRPM3 knockout mice exhibited impaired avoidance behavior in response to noxious heat. They found that temperature-dependent avoidance behavior at or above 4 5ºC in tail immersion test, and 52 ºC in hot plate test [84]. In particular, the temperature threshold in vivo was slightly higher than that observed in *Xenopus* oocyte recordings. Additionally, a temperature threshold has not been observed in DRG neurons or heterologous expression systems in mammalian cells. A similar difference between the in vitro and in vivo temperature thresholds was also reported for TRPV1 KO mice. The temperature thresholds of TRPV1 are > 42 ºC in vitro and ≥ 50 ºC in vivo (≥ 50 ºC from the tail immersion test and ≥ 52.5 ºC from the hot plate test) [9]. Although the mechanisms of temperature-dependent activation of TRPM3 await further characterization, this channel family could act as sensors of temperatures above 40 ºC. Pharmacological inhibition of TRPM3 by the TRPM3 inhibitors hesperetin, isosakuranetin, and primidone has been shown to induce prolonged latency in hot plate and tail immersion tests in mice [32, 64].

In TRPV1 knockout mice, mechanical hypersensitivity was intact, and thermal hypersensitivity caused by mustard oil and Complete Freund’s adjuvant (CFA) was reduced [9]. Similar phenotypes were observed in TRPM3 knockout mice. The avoidance of mechanical acute pain by tail clips has been observed in TRPM3 knockout mice [84]. TRPM3 knockout mice avoided the cold plate but not the hot plate [84]. TRPM3 was also found to play a role in spontaneous pain and thermal hyperalgesia during neuropathic and inflammatory pain in a carrageenan and chronic constriction injury in the sciatic nerve (CCI) model. These data revealed that TRPM3 functions in the spinal cord, central processes, and cell bodies of DRG neurons in modulating heat sensitivity [65]. TRPM3 knockout mice exhibited heat hyperalgesia but not mechanical allodynia or cold hyperalgesia in a CCI model. The heat hypersensitivity of inflammation was attenuated by treatment with the TRPM3 inhibitors isosakuranetin and primidone [32, 65].

Another work demonstrates that peripheral opioids can inhibit TRPM3 activity in sensory neurons via a Gβγ-mediated mechanism [12]. In neurons of the murine dorsal root ganglia, pro-nociceptive TRPM3 channels present in the peripheral parts of nociceptors are strongly inhibited by opioid receptor activation. Inhibition of TRPM3 channels occurs via a short signaling cascade involving Gβγ proteins, which form a complex with TRPM3 [12]. Gs-coupled Prostaglandin EP2 receptor activation inhibits TRPM3-mediated responses in sensory neurons [1]. The bradykinin BK2 receptor, a Gq-coupled receptor, inhibits TRPM3-mediated responses in sensory neurons [1]. Treatment with prostaglandin E2 or bradykinin improved CFA-induced heat hyperalgesia in the hotplate test (50 °C). The structure of mouse TRPM3 expressed in human cells with and without Gβγ analyzed by electron cryo-microscopy (Cryo-EM) demonstrated that Gβγ protein directly interact with TRPM3 structures [91]. The chemotherapeutic drug oxaliplatin is known to cause cold hypersensitivity. One study demonstrated that oxaliplatin sensitizes TRPA1 to reactive oxygen species by inhibiting prolyl hydroxylases (PHDs). Inhibition of PHD-mediated proline hydroxylation of TRPA1 sensitizes TRPA1 with cold sensitivity [43]. TRPM3 is involved in oxaliplatin-induced neuropathy. Oxaliplatin enhances TRPM3 activity and is essential for the development of oxaliplatin-induced cold and mechanical hypersensitivity [2].

A recent study has identified mutations in TRPM3 channels in pediatric patients with developmental and epileptic encephalopathies [92]. In this study, seven patients were heterozygous for the recurrent de novo TRPM3 mutation V837M (V990M), which is located in the S4-S5 linker. Another patient showed heterozygous expression of a specific TRPM3 mutation, P937Q (P1090Q), located in the outer pore region of the channel. Other reports have demonstrated that cells expressing the V990M or P1090Q mutants have higher basal calcium levels than those transfected with wild-type TRPM3 [80, 93]. Additionally, V990M and P1090Q mutants have high temperature sensitivity, that is increase in temperature from 23 to 36 °C exhibited larger increase in intracellular Ca^2+^ concentration in V990M or P1092Q expressing cells compared to that in wild-type TRPM3-expressing cells [93]. Recently, it has been suggested that the temperature of the epileptogenic region of the brain increases during epileptic seizures [61], and a link between febrile seizures caused by fever in early childhood and epilepsy has been suggested [31]. These findings indicate a relationship between brain temperature and epilepsy. There may be a relationship between the enhanced temperature sensitivity of TRPM3 and epileptic seizures.

## TRPM4

TRPM4 is a Ca^2+^-activated channel that is selective for monovalent cations and impermeable to divalent cations such as Ca^2+^ or Mg^2+^ [35, 47]. The single-channel conductance of TRPM4 is approximately 25 pS [35, 46]. The EC_50_ value for activation by Ca^2+^ varies in the whole-cell measurements between 500 nM and 20 μM. Calcium sensitivity of TRPM4 was reduced in excised inside-out patches, and dose-dependent activation was observed up to 300 μM Ca^2+^ [35, 46, 49].

Various factors modulate the sensitivity of TRPM4. PIP_2_ counteracts desensitization to intracellular Ca^2+^ in inside-out patches and rundown of TRPM4 currents in whole-cell patch-clamp experiments. Additionally, PIP_2_ increases the channel’s intracellular Ca^2+^ sensitivity 100-fold [45]. Hydrogen peroxide (H_2_O_2_) was found to eliminate in a dose-dependent manner TRPM4 desensitization. Site-directed mutagenesis experiments revealed that the Cys1093 residue is crucial for H_2_O_2_-mediated loss of desensitization [63]. Binding of calmodulin to the C-terminus of TRPM4 regulates the Ca^2+^ sensitivity of TRPM4 [49]. The Ca^2+^ sensitivity of TRPM4 is also regulated by PKC-dependent phosphorylation [49, 62]. 3,5-bis(trifluoromethyl)pyrazole (BTP2) was shown to enhance TRPM4 currents after pretreating TRPM4-expressing cells for several minutes [68]. TRPM4 activity is enhanced by the intracellular application of decavanadate, an inorganic compound with six negative charges [48]. Recently, aluminum potassium sulfate as a TRPM4 activator which does not require co-application with Ca^2+^ [54].

ATP inhibits TRPM4 channel activity [50]. Spermine, flufenamic acid, and the bitter compounds quinine and quinidine also inhibit TRPM4 [50, 70]. 9-phenanthrol has been identified as a selective TRPM4 blocker and 9-phenanthrol blocks TRPM4 with an IC_50_ of 20 μM in transfected HEK cells [18]. Glibenclamide and clotrimazole are reported to act as a TRPM4 blocker [13, 81].

In the presence of intracellular Ca^2+^, activities of TRPM4 are dramatically enhanced by temperature elevation from 15 to 35 °C. The current amplitude at + 25 mV showed a Q_10_ of 8.5, between 15 and 25 °C [69]. However, the temperature dependence of TRPM4 has not been thoroughly analyzed through electrophysiological methods.

Since the structure of TRPV1 at 3.4 Å resolution was determined using Cryo-EM in 2013 [37], the structures of many TRP channels have been elucidated. Regarding TRPM4, several groups have reported on TRPM4 with/without Ca^2+^ and TRPM4 in a lipid nanodisc [3, 86]. In 2024, Hu et al. revealed important insights into temperature-dependent structural dynamics, ligand recognition, and gating mechanisms based on a structural analysis of the temperature-sensitive TRPM4 channel at physiological temperatures using Cryo-EM [26]. The key findings include the identification of a warm conformation that is distinct from the cold conformation observed at nonphysiological temperatures. Identification of TRPM4's open state structure at physiological temperatures highlights the significance of the cold-to-warm transition in channel activation. Warm temperatures with Ca^2+^ were insufficient to open the TRPM4 channel, suggesting that some molecules are necessary for the close-to-open transition. Ligands, such as decavanadate (a positive modulator) and ATP (an inhibitor), have been shown to bind to different locations on TRPM4 at physiological temperatures compared to lower temperatures. This led to the successful observation of TRPM4 in the open state at warm temperatures. These results provide a potential molecular framework for understanding how thermo-sensitive TRPM channels perceive temperature changes.

TRPM4 is ubiquitously expressed in many tissues and has physiological functions related to circulation, immunity, cancer, and hormone secretion [14]. In excitatory cells, TRPM4 is involved in the modulation of membrane excitability and action potential. Specifically, it has been reported that knockout or pharmacological inhibition of TRPM4 shortens the firing frequency of action potentials in isolated atrial cardiomyocytes. And, pacemaker activity in the sinoatrial node results from the slow diastolic depolarization slope driven by the hyperpolarization-activated nucleotide-sensitive (HCN) channel current, Na^+^/Ca^2+^ exchange, and a Ca^2+^-activated nonselective cation current, which can be attributable in part to TRPM4 [22, 33]. TRPM4 senses increases in intracellular Ca^2+^ concentration, but its Ca^2+^ sensitivity can be maintained by a body temperature of 37 °C and by endogenous modulators such as PIP_2_. Moreover, TRPM4 is involved in chemosensory reception such as taste sensation, including sweet, umami, and bitter sensations [15]. Since sweet and umami taste sensations are enhanced at higher temperatures, the temperature sensitivity of TRPM4 could contribute to the increased perception of sweetness and umami at higher temperatures, similar to TRPM5.

## TRPM5

TRPM5 is a Ca^2+^-activated monovalent cation-permeable channel [24, 79]. Single TRPM5 channels expressed in heterologous cells show a conductance of approximately 16–25 pS [24, 39, 58]. The EC_50_ value for activation by Ca^2+^ varies in the whole-cell measurements from below 300 nM to 30 μM [24, 77].

Steviol glycosides potentiate TRPM5 activation through intracellular Ca^2+^ [1, 2]. PIP_2_ is also a cofactor of TRPM5 [39]. The Ca^2+^ sensitivity of TRPM5 is also regulated by PKC-dependent phosphorylation [62]. Calmodulin and calcium-binding protein S1 (S100A1) bind to the N-terminus of TRPM5 [7]. TRPM5 is sensitive to extracellular pH level below 7.0 and is completely blocked by pH 5.9 [40]. Extracellular zinc ion irreversibly inhibit TRPM5 activation [78]. One molecule that blocks TRPM5 is the bitter chemical quinine, which is a general inhibitor of ion channels [70]. Triphenylphosphine oxide was identified as a TRPM5 blocker [55].

Similar to TRPM4, the activity of TRPM5 was dramatically enhanced by increasing the temperature from 15 to 35 °C in the presence of intracellular Ca^2+^. The Q_10_ value of the TRPM5 current with intracellular Ca^2+^ was 10 [69].

The expression of TRPM5 channels is restricted to taste cells, the pancreas, brainstem, olfactory epithelium, and olfactory nerves, where they are involved in controlling membrane potentials [38]. Some reports have indicated that TRPM5 has physiological functions associated with insulin secretion [11] and signaling of tastes [69]. In pancreatic β-cells, it is reported that TRPM5 modulates glucose-induced Ca^2+^ oscillation. Like TRPM4, while TRPM5 might sense increases in intracellular Ca^2+^ concentration and modulate membrane potential, Ca^2+^ sensitivity of TRPM5 can be maintained by a body temperature of 37 °C [11]. Within taste cells (type II taste cells), TRPM5 is the final element in a signaling cascade that starts with the activation of G protein-coupled receptors by bitter, sweet, or umami taste molecules, which requires phospholipase C (PLC). PLC hydrolyzes PIP_2_ into diacylglycerol (DAG), inositol 1,4,5-triphosphate (IP_3_), and IP_3_ causing an increase in the intracellular calcium concentration through the release of Ca^2+^ from intracellular stores, followed by the activation of TRPM5. Membrane depolarization caused by TRPM5 activation results in ATP release [27]. TRPM5 is required for normal taste, as TRPM5 knockout mice are dramatically less sensitive to bitter, sweet, and umami tastes, although they retain their ability to detect sour and salty tastes [90]. Banik et al. demonstrated that both TRPM4 and TRPM5 are required for normal signaling in taste receptor cells by analyzing TRPM4 and TRPM5 double-knockout mice [15]. The temperature sensitivity of both TRPM4 and TRPM5 may contribute to temperature-dependent taste reception. At least, Talavera et al. demonstrated that increasing temperature between 15 and 35 °C markedly enhanced the gustatory nerve response to sweet compounds in wild-type mice but not in TRPM5 knockout mice [69]. Moreover, TRPM5 could also be an element in the transduction of fat and high-salt tastes [41, 53].

TRPM5 is also expressed in motor neurons. Bistable motor neurons can maintain two distinct stable electrical states (activity states) depending on the stimuli or conditions. Bistable motoneurons of the spinal cord exhibit a warmth-activated plateau potential driven by Na + and triggered by brief excitation. TRPM5 is the main molecular player in the bistability of mouse motoneurons. In the motoneurons, TRPM5 is activated by Ca^2+^ released via the ryanodine receptor. Silencing TRPM5 in motoneurons leads to hind limb paresis and causes difficulties in executing high-demand locomotor tasks [6].

## Perspectives

As described in this review, TRPM3 plays a significant role in temperature-related physiological processes, such as heat nociception. In contrast, although TRPM4 and TRPM5 exhibit temperature sensitivity, their specific physiological roles in response to temperature remain largely unclear. At least, these channels expressed in tissues not exposed to dynamic temperature changes can show high sensitivity to Ca^2+^ at core body temperature. Temperature changes could modulate signal transduction through TRPM4 and TRPM5, as well as the sensitivity to activating stimuli for TRPM4 and TRPM5. Further analysis of these channels is expected to reveal temperature-related biological phenomena and deepen our understanding of the importance of temperature sensation and the associated physiological functions.

## Data Availability

Not applicable.
